# Muscle oxygenation trends after tapering in trained cyclists

**DOI:** 10.1186/1476-5918-4-4

**Published:** 2005-03-24

**Authors:** J Patrick Neary, Donald C McKenzie, Yagesh N Bhambhani

**Affiliations:** 1 Faculty of Kinesiology, University of New Brunswick, Fredericton, New Brunswick, Canada; 2 Faculty of Human Kinetics, Allan McGavin Sports Medicine Centre, University of British Columbia, Vancouver, British Columbia, Canada; 3 Faculty of Rehabilitation Medicine, Department of Occupational Therapy, University of Alberta, Edmonton, Alberta, Canada

## Abstract

**Background:**

This study examined muscle deoxygenation trends before and after a 7-day taper using non-invasive near infrared spectroscopy (NIRS).

**Methods:**

Eleven cyclists performed an incremental cycle ergometer test to determine maximal oxygen consumption (VO_2_max = 4.68 ± 0.57 L·min^-1^) prior to the study, and then completed two or three high intensity (85–90% VO_2_max) taper protocols after being randomly assigned to a taper group: T30 (n = 5), T50 (n = 5), or T80 (n = 5) [30%, 50%, 80% reduction in training volume, respectively]. Physiological measurements were recorded during a simulated 20 km time trials (20TT) performed on a set of wind-loaded rollers.

**Results and Discussion:**

The results showed that the physiological variables of oxygen consumption (VO_2_), carbon dioxide (VCO_2_) and heart rate (HR) were not significantly different after tapering, except for a decreased ventilatory equivalent for oxygen (V_E_/VO_2_) in T50 (p ≤ 0.05). However, during the 20TT muscle deoxygenation measured continuously in the vastus medialis was significantly lower (-749 ± 324 vs. -1140 ± 465 mV) in T50 after tapering, which was concomitant with a 4.53% improvement (p = 0.057) in 20TT performance time, and a 0.18 L·min^-1 ^(4.5%) increase in VO_2_. Furthermore, when changes in performance time and tissue deoxygenation (post- minus pre-taper) were plotted (n = 11), a moderately high correlation was found (r = 0.82).

**Conclusion:**

It was concluded that changes in simulated 20TT performance appeared to be related, in part, to changes in muscle deoxygenation following tapering, and that NIRS can be used effectively to monitor muscle deoxygenation during a taper period.

## Background

Near infrared spectroscopy (NIRS) is a non-invasive optical technique that is based on the differential absorption properties of hemoglobin (Hb) and myoglobin (Mb) in the near infrared (700–1000 nm) range. During the last decade, NIRS has been used extensively to measure muscle oxygenation both qualitatively [1-3] and quantitatively [4-7] during exercise. NIRS has become an appealing research tool due to its non-invasive nature for the measurement of localised blood volume and oxygenation. In these exercise studies, NIRS has been used to estimate the lactate threshold [8,9], to predict [10] and objectively [11] measure the ventilation threshold, to reflect exercise intensity [8,12], and to monitor muscle [13] and blood [14] metabolites. Validation [6,15,16] and reliability [1,2,17] studies have also been undertaken, and a theoretical model for muscle oxygenation (i.e., oxidative metabolism) measured by NIRS has been presented [18]. More recently, NIRS has been used to examine the effects of short-term endurance training on muscle oxygenation in a group of healthy untrained subjects [19], and competitive well-trained cyclists [20]. These studies showed that NIRS was a viable non-invasive technique to monitor muscle oxygenation, and to reflect the physiological adaptations in peripheral skeletal muscle following a short-term endurance-training program.

Tapering is a training method performed by athletes during the days (3-21d) immediately prior to competition to create a "rebound" effect to improve performance. Physiological adaptations have also been reported during a period of tapering [21-26]. Both central cardiovascular (i.e., VO_2_max) and peripheral muscular (i.e., oxidative enzyme) changes have been documented and are correlated with improvements in performance following the taper period. It is likely that the enhanced oxidative capacity following tapering would elicit significant changes in muscle oxygenation. However, this has not been tested prospectively. The focus of this pilot study (due to the limited sample size in each group) was to test the hypothesis that muscle oxygenation changes, measured by NIRS, will occur following a period of tapering in a group of trained competitive cyclists. Furthermore, the possibility exists that NIRS can be used to monitoring the training status of an athletic and/or non-athletic population. Having a non-invasive technique that allows one to monitor peripheral changes and training adaptations occurring in the local muscle tissue will have a great impact on the understanding of local muscle tissue metabolism and haemodynamics.

## Methods

### Subjects

These data are based on eleven competitive male cyclists between the ages of 19–34 yr that volunteered to participate in this study. Some of these subjects completed more than one taper, explaining why each group had an equal number of five subjects per group. Two subjects completed all three tapers, while three subjects completed two of the tapers. Physical characteristics for the eleven subjects were: age (Mean ± SD) = 22.6 ± 4.7 yr, height = 177.0 ± 5.0 cm, body mass = 70.3 ± 5.0 kg, thigh skinfold thickness = 9.0 ± 1.8 mm, VO_2_max = 4.68 ± 0.57 L·min^-1 ^(66.5 mL·kg^-1^·min^-1^). Each subject completed a written informed consent after a thorough explanation of the protocols and procedures involved in accordance with an institutional ethics review committee.

### Testing

Prior to the taper phase, each subject completed an incremental VO_2_max test on a calibrated Monark cycle ergometer (Model 818E, Varberg, Sweden) as described previously [27]. Following a two minute rest period while sitting stationary on the cycle ergometer, each cyclist began pedalling at an initial work rate of 80 watts (W) for 2 min, followed by 45 W increments every minute up to 260 W. Thereafter, work rate was increased each minute by 20 W increments to volitional fatigue. This test lasted approximately 10–14 min in duration. Expired gases were collected and analysed by open circuit spirometry by using an automated metabolic analysis system (Vmax, Sensormedics, California, USA). The data were averaged in 20 sec intervals. The gas analysers were calibrated with primary standard gas (16.0% O_2_, 4.0% CO_2_, balance N_2_) before and after each individual test. The pneumotach was calibrated using a 3L syringe. The following criteria were used to verify VO_2_max: a respiratory exchange ratio (RER) greater than 1.12, and an increase in VO_2 _uptake less than 100 mL with an increase in work rate [28]. All subjects achieved these criteria. Heart rate (HR) was monitored continuously by telemetry (Polar monitors, Electro, Finland).

Because of the endurance characteristics of these cyclists (both road and mountain bike), a simulated 20TT performance ride was used as the criterion test (i.e., index of performance) before and after tapering to evaluate the physiological and performance effects of each taper protocol. Each cyclist was asked to complete the ride as fast as possible, with no feedback provided on how well he was performing until the end of the test. The pre-taper 20TT was used as the last workout prior to tapering. The description of this test has been reported in detail previously [27,29]. Briefly, each cyclist used his own bicycle mounted on a set of aluminium cast wind-loaded cycling rollers fitted with a stabilizing bar that was attached to the handle bar of the bicycle for safety. The air pressure of the tires was checked before and after each ride to ensure that maximum pressure was maintained. The same set of cycling rollers was used for all simulated 20TT rides with the rollers being interfaced with a computer to record velocity, distance, and cycling time. This device was calibrated by measuring the circumference of the rollers (and thus the distance was a product of the circumference and rpm, which was recorded by the computer [29]). Respiratory gas exchange responses (averaged over 20 sec) were monitored for 2–3 min every 5 km during the pre-and post-taper simulated 20TT. The same calibration procedures were performed on the gas analysers as previously stated above for the VO_2_max test. HR was monitored continuously throughout the duration of the 20TT using telemetry (Polar, Finland). The Rating of Perceived Exertion (RPE) was also taken at the end of each work rate throughout the VO_2_max test, and during each 5 km interval using the Borg Scale [30].

### Taper Protocols

The experimental design for this study is illustrated in Table 1. A 3-week high-intensity endurance-training period preceded the taper for consistency in training volume between cyclists. This involved indoor stationary cycling (Blackburn Magnetic Turbo Trainer, USA) 4 sessions·wk^-1^, for 60 minutes·session^-1 ^at 85–90% VO_2_max. Heart rate was monitored for all cycling rides using telemetry, and each ride was supervised to ensure that the cyclist maintained their required training intensity. Prior to this consistency period, these cyclists were road training approximately 150–250 km·wk^-1^. Before the 7-day taper began, each cyclist was randomly assigned to one of three taper protocols: **T30 **= 30% reduction in weekly training volume (n = 5), **T50 **= 50% reduction in weekly training volume (n = 5), **T80 **= 80% reduction in weekly training volume (n = 5). However, for those cyclists that completed more than one taper protocol, a 3-week baseline training period was implemented to avoid any interference between tapers [31]. Training volume in this study was defined as a combination of duration and frequency (see Table 1).

**Table 1:** Experimental design for the 7-day (DO to D7) taper protocols. Time is expressed in minutes, and denoted in the table with an apostrophe ('); 20TT = simulated 20 km time trial performance ride.

**TAPER**	**DO**	**D1**	**D2**	**D3**	**D4**	**D5**	**D6**	**D7**
**T30 (n = 5)**	20TT	Rest	60'	55'	50'	45'	Rest	20TT
**T50 (n = 5)**	20TT	Rest	45'	40'	35'	30'	Rest	20TT
**T80 (n = 5)**	20TT	Rest	30'	15'	10'	5'	Rest	20TT

### Near Infrared Spectroscopy (NIRSCWS)

A continuous dual wavelength spectrometer (NIR_CWS_) (RunMan, NIM Inc., PA, USA) was used to measure tissue absorbency (Hb/Mb-O_2_; mV) of the right vastus medialis muscle at 760 nm and 850 nm in each subject during the 20TT. The procedures and protocols involving the use of the NIR_CWS _during this experimentation have been reported previously [3,27]. Briefly, the NIR_CWS _probe was calibrated at 760 nm and then 850 nm according to the manufacturer's protocol and specifications prior to each test. This was done while the subject was seated on his bicycle with the leg in a relaxed position at the lowest point of the pedal. The electrical output of the NIR_CWS _unit was adjusted by initially setting the balance at 0 ± 10 mV and then adjusting the gain control to read between 600 to 1000 mV at 760 nm. The absorbency measurements were then checked at 850 nm to ensure that the values were negative in value and within 10% of those observed at 760 nm. A settling time of approximately 20 sec was allowed at each wavelength to allow for stability of the readings. The calibration was repeated twice before each test to ensure accuracy of the data. In this study the NIR_CWS _unit was interfaced with a computer as previously described [2,11] with the data viewed on-line during each test. The inter-session reliability of the minimum deoxygenation value for this muscle group during cuff ischaemia in this laboratory is 0.95 [2].

To record the NIR_CWS _signal, the probe was placed over the right vastus medialis muscle, approximately 14–20 cm from the knee joint along the vertical axis of the thigh [1]. At this time, a thigh skinfold measurement (Harpenden Skinfold Calipers) was also taken to record subcutaneous adiposity to ensure that skinfold thickness was not a confounding variable for the measurement of muscle oxygenation (Table 2). After measuring and locating the desired anatomical position, a permanent ink mark was placed on the leg of each cyclist, and recorded for subsequent post-taper NIR_CWS _measurements. A piece of clear plastic wrap was placed around the probe to cover the photo-detectors to prevent distortion of the signal caused by sweating during exercise. The signal was recorded at a depth of approximately 1.5 cm (i.e., separation distance between the emitter and detector sensors was 3 cm), and at sampling frequency of 30 Hz using an A/D board and customized software interfaced with the computer [11]. Measurements were undertaken continuously for two minutes at rest (i.e., baseline value), throughout exercise (25–30 minutes) and for six minutes of recovery. During the first two minutes of the recovery period the subject pedalled their bicycle at a comfortable intensity (active recovery), while during the remaining four minutes the cyclist sat on the bicycle with the right foot in a relaxed position at the bottom of the pedal (passive recovery). The raw NIR_CWS _signal (mV) was used to reflect the trend in tissue oxygenation, and this method has been reported previously in the literature [20,27]. Furthermore, both the oxy-Hb (850 mV) and the deoxy-Hb (760 mV) signals were examined from this set of data. These signals demonstrated an opposite trend, indicating that changes in the oxygenation (Hb/Mb-O_2_) and deoxygenation (Hb/Mb) most likely reflect changes in oxygen extraction. Therefore, the Hb/Mb-O_2 _signal was used to represent the findings from this study. This signal was averaged every 20 seconds to correspond with the metabolic measurements. For comparison of the NIR_CWS_data before and after tapering, a delta score was calculated by subtracting the initial resting baseline value (averaged over 2 minutes of rest) from each 20 second averaged value throughout the complete test [20]. This subtraction eliminates basal muscle metabolism [18] and allows a direct comparison before and after tapering, and among individuals.

**Table 2:** Physiological responses during the simulated 20 km time trial (20TT) Pre- and Post-Taper. Values are Mean (SD). **T30 **(30% reduction in weekly training volume; n = 5), **T50 **(50% reduction in weekly training volume; n = 5), **T80 **(80% reduction in weekly training volume; n = 5). * indicates significantly different from Pre-test, p ≤ 0.05; #indicates p = 0.057 from Pre-test.

**Variable**	**Pre-Taper**			**Post-Taper**		
	**T30**	**T50**	**T80**	**T30**	**T50**	**T80**

Time (min)	27:03(1:29)	**26:34(0:56)**	26:10(2:58)	27:40(2:34)	**25:25(1:23)#**	25:31(2:07)
VO_2 _(L·min^-1^)	3.28 (0.54)	4.04 (0.24)	4.09 (0.59)	3.17 (0.56)	4.22 (0.11)	4.15 (0.39)
%VO_2_max	74.6%	86.9%	84.7%	72.1%	90.7%	85.3%
VCO_2_(L·min^-1^)	3.39 (0.95)	3.82 (0.26)	3.84 (0.49)	3.31 (0.93)	4.07 (0.21)	4.07 (0.31)
V_E _(L·min^-1^)	81.0 (17.2)	102.1 (12.2)	103.1(13.2)	81.7 (16.4)	95.0 (5.9)	106.1 (10.2)
V_E_/VO_2_	24.7 (1.9)	**25.3 (2.2)**	25.2 (1.1)	25.8 (1.0)	22.5 (1.7) *	26.1 (1.2)
RER	1.03 (0.07)	0.95 (0.02)	0.94 (0.03)	1.04 (0.03)	0.97 (0.04)	0.98 (0.03)
HR (b·min^-1^)	169 (5)	175 (9)	173 (13)	173 (4)	174 (13)	179 (5)
O_2 _pulse (mL·b^-1^)	19.3 (2.8)	23.2 (2.4)	23.6 (1.8)	18.3 (3.3)	24.3 (2.2)	21.9 (1.4)
Hb/Mb-O_2 _(mV)	-455 (270)	**-731 (155)**	-770 (183)	-403 (170)	-1167 (224) *	-847 (106)
RPE	13.9 (2.3)	16.2 (1.6)	16.3 (1.3)	14.9 (2.0)	16.8 (1.5)	16.3 (1.2)
Thigh Skinfold (mm)	8.9 (1.8)	9.2 (1.7)	9.0 (1.9)	9.0 (1.9)	9.1 (1.6)	9.2 (1.8)

### Statistic analysis

An analysis of variance model (ANOVA) was used initially to determine the homogeneity of variance of the data. A Levine's test (on absolute deviations) for Group × NIRS was violated (df = 2,387; F = 17.11; p = 0.000), and therefore a Mann-Whitney U non-parametric test was used to determine significant differences between pre- and post-taper trials. For the purpose of this study, the alpha level for significance was set at p ≤ 0.05. All statistical analyses were performed using Statistica 6.0 software program (Stats Soft, Tulsa OK, USA). All values are expressed as Mean ± SD. A significant Group main effect was apparent, but post hoc analysis revealed non-significant means pre- to post-taper for all dependent (cardiovascular) variables measured except for a significant different V_E_/VO_2 _ratio in the T50 group.

## Results

### Cardiovascular responses

All subjects attained their individual VO_2_max according to the criteria established (see Methods). The average exercise intensity during the 20TT was between 72% and 90% VO_2_max. Table 2 illustrates the physiological responses of these cyclists during the simulated 20 km time trials before and after tapering, showing that a significantly lower V_E_/VO_2 _ratio (25.3 ± 2.2 to 22.5 ± 1.7; p ≤ 0.05) occurred in the T50 group. Performance time was improved on average by 1:09 min:sec in the T50 group (26:34 ± 0:56 to 25:25 ± 1:23; p = 0.057), but unchanged in the other groups (Table 2).

### Muscle Oxygenation Trends

**Figure 1:**
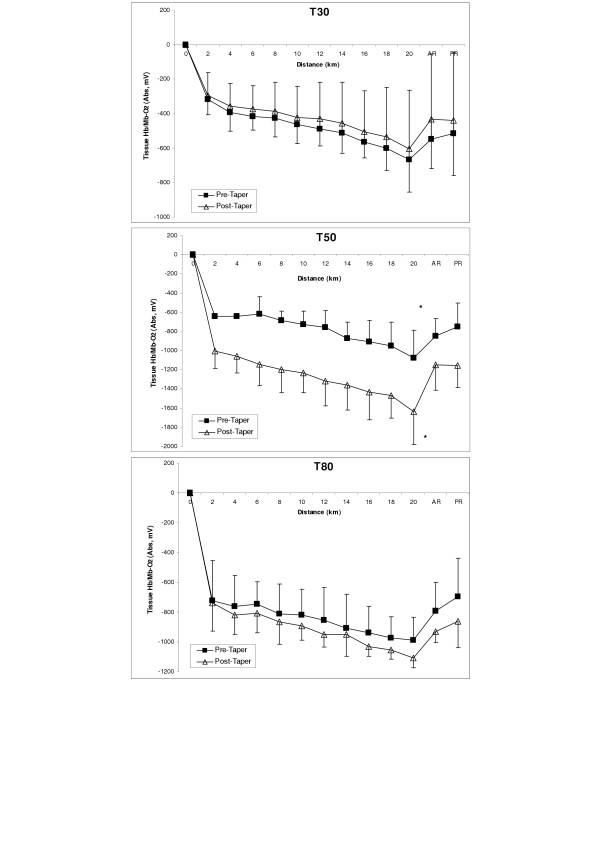
Group mean (± SD) tissue oxygenation (Hb/Mb-O_2_; Absorbency, mV) during the simulated 20 km time trial performance ride Pre- and Post-taper in the three taper groups. Panel **T30 **(30% reduction in weekly training volume; n = 5); Panel **T50 **(50% reduction in weekly training volume; n = 5); Panel **T80 **(80% reduction in weekly training volume; n = 5). * denotes significance (p ≤ 0.05) between pre- vs. post-taper trials.

The trend in mean muscle oxygenation (Hb/Mb-O_2_) during the 20TT before and after the taper for each group is shown in Figure 1. A consistent pattern in tissue deoxygenation was found for all performance rides. Tissue Hb/Mb-O_2 _showed a rapid decrease during the first 2 km of the simulated race, followed by a gradual decline or plateau until the end of the race (range = 25 to 30 minutes). Mean tissue oxygenation values for the complete test (Table 2) was not different (U = 2038, z = -0.347, p ≥ 0.05) for the T30 protocol (-455 ± 270 to -403 ± 170 mV), and the T80 (U = 1691, z= 1.96, p ≥ 0.05) protocol (-770 ± 155 to -847 ± 106 mV), but was significantly greater (i.e., increased muscle deoxygenation) following the 50% reduction (T50) training volume taper (U = 642, z = 6.85, p ≤ 0.05) protocol (-731 ± 155 to -1167 ± 224 mV). It was interesting to note that the T50 group had a decreasing (p ≤ 0.05) trend in tissue Hb/Mb-O_2 _with a concomitant 0.18 L·min^-1 ^(4.5%) increase in oxygen consumption after tapering during the 20TT.

**Figure 2:**
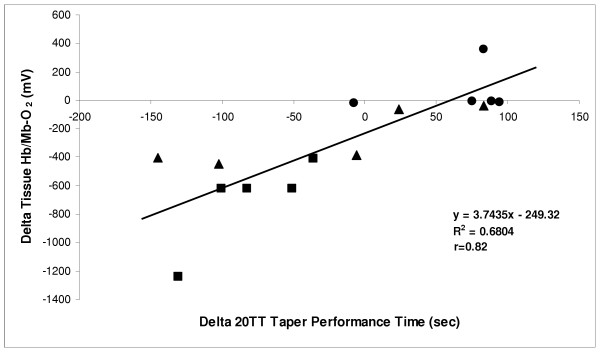
Relationship (r = 0.82) between the change (delta) in Tissue Hb/Mb-O_2 _(mV) vs. 20TT Taper Performance time (minutes) for all subjects combined (n = 11) after tapering. Note that a negative value for performance time indicates an improvement in performance (i.e., a faster 20TT ride). Delta scores were determined by post-taper minus pre-taper values. Each symbol represents a different taper group (● T30; ■ T50; ▲ T80).

All three groups of cyclists also showed a delay in tissue Hb/Mb-O_2 _during the recovery phase following the 20TT ride. In fact, muscle oxygenation did not return to baseline before or after tapering, and remained well below baseline during this recovery phase (Figure 1). In general, the trend in muscle deoxygenation followed the metabolic response of each cyclist during the simulated ride. Also, it was interesting to note that there was a moderately high correlation (r = 0.82) between the change in tissue Hb/Mb-O_2 _vs. 20TT taper performance time when all cyclist were combined (Figure 2). The delta value was calculated by post-taper minus pre-taper score.

## Discussion

The results of the present study are the first to show that peripheral adaptations in muscle oxygenation can occur with a proper preparation period prior to competition (i.e., taper), and that these adaptations can elicit improvements in performance. For example, the T50 protocol resulted in a significantly greater muscle deoxygenation after the taper with a concomitant increase in O_2 _uptake (4.5%) and an improvement (4.53%) in performance (Table 2). It was also interesting to note that when the change in tissue Hb/Mb-O_2 _was plotted against the change in 20TT taper performance time for all subjects combined (n = 11), a moderately strong correlation was found (Figure 2; r = 0.82). This suggests that metabolic changes that occur in the vastus medialis muscle are, in part, responsible for changes in performance. The decrease in oxygen levels presented in this paper is consistent with an increase in oxygen extraction (i.e., more oxygen uptake for a given blood flow). Previous studies have shown both oxygen delivery and oxidative metabolism to improve with training [19,32], and now, presumably with a taper. This is consistent with previous research on tapering which showed that changes in oxidative enzyme activity (cytochrome oxidase) correlated (r = 0.84) with changes in 40 km time trial performance time [33].

As indicated earlier, the intent of this study was to examine the effects of tapering on muscle oxygenation using NIR_CWS_, and not to test the differences between groups per se. One limitation of this study was the small sample size in each of the taper groups (n = 5). Notwithstanding this limitation, it was still evident from these data that NIR_CWS _successfully measured the trends in muscle oxygenation during the three different taper protocols. Using non-parametric statistics (Mann-Whitney U), NIR_CWS _demonstrated that the T50 protocol (50% reduction in weekly training volume) resulted in a significantly greater muscle deoxygenation (i.e., a difference in the balance between O_2 _delivery and utilisation) after the 7-day linear taper. This increase in muscle deoxygenation resulted in a concomitant increase of 0.18 L·min^-1 ^of O_2 _uptake during the time trial for the T50 group, which translated into a 4.53% improvement in 20TT performance time (26:34 ± 0:56 to 25:25 ± 1:23 min:sec; p = 0.057).

A number of physiological adaptations to tapering have been reported in the literature. For example, the oxidative capacity of the muscle is enhanced as a result of an increase in oxidative enzyme activity [21,31,33]. As well, an elevated muscle glycogen concentration [21,31], and, improved haematological and biochemical profiles following a period of tapering in competitive athletes [23,34] have been documented. More recently, Trappe et al. [35] showed physiologic changes in single muscle fibres after tapering. Furthermore, tapers in which a systematic step-wise reduction in training volume was maintained at high exercise intensity showed greater improvements in performance [22,24,31,36]. Thus, the results of the present study also support the contention that peripheral adaptations can occur with a proper preparation period prior to competition (i.e., taper), and can elicit improvements in performance.

Physiologically, acute changes in tissue Hb/Mb-O_2 _are related to changes in muscle blood flow, O_2 _extraction and venous return [1]. An increased O_2 _extraction [i.e., arterio-venous O_2 _difference; (a-v)O_2diff_] during the taper is most likely the result of an increase in oxidative enzyme activity, and/or an improvement in the haematological parameters, both of which have been shown to improve with tapering [21,23,31]. The (a-v)O_2__diff _itself can be affected by the rate of diffusion of oxygen from the blood into the myocyte, the [Hb], and by the oxyhemoglobin dissociation curve. It is reasonable to assume that the significant increase in muscle deoxygenation in the T50 group after the 7-day taper period in the present study was related to the above mentioned cellular and biochemical changes. An increased (a-v)O_2diff _results because of the structural adaptations in skeletal muscle in response to alterations in metabolic demand. However, a second limitation of this study is that we do not have muscle biopsy or blood data to confirm our hypothesis. Albeit, the physiological consequence of these peripheral adaptations would mean that the working skeletal muscle can extract greater amounts of O_2_, which in turn should allow for an improved endurance performance [37]. Endurance performance on the 20TT for the T50 group reached a significance level of p = 0.057 when comparing the pre- to post-taper test results, although previous research in our laboratory has demonstrated that 20TT performance was significantly increased in a slightly larger group (n = 6) of well-trained cyclists when using a similar protocol [38]. Furthermore, research has demonstrated that skeletal muscle adaptations (i.e., increased oxidative capacity and mitochondrial activity) can coincide with positive changes in performance following a taper period [21,31,35]. However, it is unknown whether the changes in muscle deoxygenation measured by NIRS_CWS _correlate with changes in oxidative enzymes during the taper, although Puente-Maestu et al. [32] showed that changes in the recovery deoxygenation signal (τHbO_2_) correlated (r = 0.76) with changes in citrate synthase enzyme activity in muscle biopsy tissue.

Another possibility for the increased deoxygenation during the taper maybe related to increases in blood flow to the skeletal muscle. Green et al. [39] speculated that alterations in blood flow might be one of the earliest adaptive responses to training, and possibly capable of eliciting the same physiologic advantage as the increase in the muscles enzymatic machinery. Research is available to demonstrate that increases in plasma volume and red blood cell volume occur after tapering [40]. This would allow a greater blood flow to be available for the contracting muscle post-taper. Unfortunately, we do not have this data available to confirm this hypothesis. Therefore, whether the increased muscle deoxygenation from pre- to post-taper (T50) is related to the alteration in muscle blood flow, and simultaneous increases in oxidative enzymes and blood (cell) volume, is yet to be determined. It is now possible using spatially resolved spectroscopy (NIRS-SRS) to examine the contribution of blood volume. Research has also shown that changes in blood lactate, both acute [8,9], and chronic [19], can also influence the NIR_CWS _signal during exercise.

The data from this study is also important as it reflects individual cyclists' responses to each particular taper protocol. For example, in the T50 protocol four of the five cyclists demonstrated significant muscle deoxygenation trends after tapering. As well, an examination of the T80 individual results showed that four of the five cyclists demonstrated this decline in muscle oxygenation following tapering although no significant difference occurred post-taper in the T80 group. Future research is warranted to determine the reason(s) for this. In general, those in the T50 and T80 taper protocols showed a greater muscle deoxygenation, which coincided with their personal changes in performance and physiological (VO_2_, VCO_2_, V_E_, HR, RER) measurements (also see Figure 2). A closer examination of Figure 2 illustrates that the T50 group had faster performance times and a great deoxygenation. However, four of the five cyclists in the T30 taper protocol showed the opposite effect (i.e., greater muscle oxygenation and an unchanged or slower performance time). These results would suggest that the minimal 30% reduction in weekly training volume during the taper was not enough to elicit physiological or performance adaptations, possibly due to residual fatigue accompanying the excessive training during the taper phase. This is consistent with the philosophy that sufficient rest is needed to express the adaptations concomitant with training, and, supports previously published literature that optimal amounts of rest and exercise are needed during the taper [21,24-26,31,33].

The improved performance time during the simulated 20TT in the T50 taper group was consistent with the decreased tissue Hb/Mb-O_2 _(NIR_CWS_) data (-731 ± 155 to -1167 ± 224 mV; p ≤ 0.05). This supports recent work by Nioka et al. [41] and Bae et al. [8] who showed that the degree of muscle deoxygenation varies in accordance with the intensity of exercise and the level of training. Thus, it appears that NIR_CWS _was sensitive enough to detect the changes in muscle oxygenation following the different taper protocols, and NIR_CWS _is a viable non-invasive technique to monitor muscle oxygenation in peripheral skeletal muscle following a taper period. Puente-Maestu et al. [32] concluded that "NIRS may be a useful non-invasive tool for detecting the adaptations of skeletal muscle to training".

Another important observation from this data was that muscle re-oxygenation during recovery from the prolonged high-intensity exercise during the 20TT was delayed and did not return to baseline after 6 minutes of (active and passive) recovery. Although some reports have shown that Hb/Mb-O_2 _resaturation is not affected by pH when using small muscle mass [42], a delayed re-oxygenation has been shown in highly oxidative muscle following isometric contractions [43]. However, these conditions are different from the current study which used whole body exercise and involved a greater muscle mass (i.e., vastus medialis). Im et al. [44] showed that muscle desaturation was related (r = 0.83) to whole body VO_2 _during dynamic exercise involving a large muscle mass. Therefore, taken together, these data suggest that a number of possible factors may be influencing this re-oxygenation including, but not limited to, muscle fibre type, type of muscular contraction and mode of exercise, capillary recruitment immediately post exercise, a high intramuscular temperature, and the presence of muscle metabolites (i.e., [H^+^], increased lactic acid). Further research is needed to identify the relative contribution of those factors that are associated with the delayed recovery as measured by NIRS.

## Conclusion

In summary, these results are somewhat unique and suggest that a positive adaptation to taper training is that blood volume and metabolic changes lead to an increase in O_2 _extraction. Furthermore, that increased oxygen extraction is associated with increased performance. Therefore, it appears that NIRS_CWS _can be used to record the peripheral metabolic changes in trained endurance cyclists after a period of taper training, and this research has contributed further to our understanding of the application of NIRS to exercise sports science [45-47].

## Authors' contributions

JPN and DCM conceived the study and wrote the research grant; YNB participated in its design, coordination and provided the NIRS equipment and expertise; JPN and YNB carried out the testing and data analysis of the study. All authors read and approved the final manuscript.
